# Snake Optimization Algorithm Augmented by Adaptive *t*-Distribution Mixed Mutation and Its Application in Energy Storage System Capacity Optimization

**DOI:** 10.3390/biomimetics10040244

**Published:** 2025-04-16

**Authors:** Yinggao Yue, Li Cao, Changzu Chen, Yaodan Chen, Binhe Chen

**Affiliations:** School of Intelligent Manufacturing and Electronic Engineering, Wenzhou University of Technology, Wenzhou 325035, China; yyg@wzut.edu.cn (Y.Y.); chenchangzu@163.com (C.C.); elevenward@sina.cn (Y.C.); chenbinhe@126.com (B.C.)

**Keywords:** snake optimization algorithm, tent chaotic map, reverse learning, adaptive *t*-distribution mutation, engineering application problems

## Abstract

To address the drawbacks of the traditional snake optimization method, such as a random population initialization, slow convergence speed, and low accuracy, an adaptive *t*-distribution mixed mutation snake optimization strategy is proposed. Initially, Tent-based chaotic mapping and the quasi-reverse learning approach are utilized to enhance the quality of the initial solution and the population initialization process of the original method. During the evolution stage, a novel adaptive *t*-distribution mixed mutation foraging strategy is introduced to substitute the original foraging stage method. This strategy perturbs and mutates at the optimal solution position to generate new solutions, thereby improving the algorithm’s ability to escape local optima. The mating mode in the evolution stage is replaced with an opposite-sex attraction mechanism, providing the algorithm with more opportunities for global exploration and exploitation. The improved snake optimization method accelerates convergence and improves accuracy while balancing the algorithm’s local and global exploitation capabilities. The experimental results demonstrate that the improved method outperforms other optimization methods, including the standard snake optimization technique, in terms of solution robustness and accuracy. Additionally, each improvement technique complements and amplifies the effects of the others.

## 1. Introduction

Many different sectors are faced with an endless variety of challenging optimization challenges in the current context of fast technological growth. The development of swarm intelligence optimization algorithms has flourished due in large part to these circumstances [1]. From an engineering perspective, the layout design of large-scale integrated circuits requires that numerous components be arranged in a limited chip space while meeting tight requirements, including signal transmission and heat dissipation. The optimization of aircraft forms in aeronautical engineering is linked to several metrics, including stability, energy consumption, and flying efficiency. Intricate restrictions and a large number of design variables are involved in this undertaking [2]. Even a small change in the scheduling of the production process in industrial manufacturing can set off a series of events, including delivery delays and resource idleness. When dealing with high-dimensional, nonlinear, non-differentiable, and even dynamically changing objective functions and restrictions, traditional optimization techniques that rely on exact mathematical models and gradient information are inadequate. These algorithms’ computing complexity increases exponentially with the magnitude of the problem, and they rely heavily on precise mathematical representations of the issue [3]. Once they encounter practical problems lacking explicit expressions, they are at a loss as to how to proceed.

Swarm intelligence optimization methods have become quite important in this context. The intelligent behaviors of real biological populations, such fish schooling, bird migration, and ant colony feeding, are mimicked by these algorithms. They do not necessitate a thorough comprehension of the intricate interior workings of an issue. Instead, they can discover roughly optimum solutions in complicated and changing situations based on just basic individual behavior norms, interactions, and collaboration among people. This not only demonstrates outstanding robustness and adaptability, allowing for rapid adaption to various conditions, but it also loosens the stringent requirements for prior knowledge. Consequently, it significantly decreases the time it takes to find a solution, lowers computing expenses, and creates new opportunities for the academic and industrial sectors to address difficult issues. The advantages have been felt in many industries. For example, the ant colony method is used in distribution and logistics to optimize routes and save on transportation costs. The particle swarm optimization technique is used in the power system to optimize reactive power and preserve grid stability [4]. The snake-shaped optimization method has emerged within the “family” of swarm intelligence optimization algorithms. The way snakes seek and survive in the wild serves as an inspiration. Snakes rely on their keen senses, adaptable movement, and teamwork to seek prey in challenging environments and avoid obstacles [5]. This trait is condensed into a special optimization technique by the snake-shaped optimization algorithm: snake-shaped people move and “swim” around the search space, constantly changing their routes based on peer locations and environmental information. In the early stages, they daringly explore, and in the later stages, they perfectly converge. This method approaches the ideal answer due to the group’s very effective information exchange and combined efforts [6]. It has distinct advantages over other conventional swarm intelligence algorithms in that it avoids local optima and strikes a balance between exploration and exploitation. It provides fresh viewpoints and effective solutions for resolving cutting-edge issues including dynamic environment optimization and multimodal function optimization. The theoretical depth and application range of swarm intelligence optimization algorithms are constantly being enhanced and broadened.

### 1.1. Problem Statement and Motivation

Swarm intelligence algorithms, characterized by their unique population collaboration mechanisms and search strategies, offer novel approaches to solve complex optimization problems. They hold remarkable application value across diverse fields. Currently, as the scale of engineering projects and the complexity of data continue to increase, traditional optimization algorithms face challenges such as slow convergence and a tendency to become trapped in local optima when dealing with large-scale high-dimensional problems. Research on swarm intelligence optimization algorithms can not only enrich the theoretical framework of optimization algorithms and deeply analyze their search performance and convergence characteristics in different scenarios, but also provides a theoretical basis for solving practical engineering problems. By leveraging swarm intelligence optimization algorithms, we can enhance the prediction accuracy and generalization ability of machine learning models while tuning their parameters, reducing computational costs. In resource scheduling scenarios, these algorithms enable efficient resource allocation, contributing to cost reduction and efficiency improvement for enterprises. At the moment, metaheuristics and mathematical programming are the two primary categories of algorithmic optimization techniques that are in use [7]. Mathematical programming encompasses traditional mathematical approaches like mathematical programming and integer programming, which are challenging to use in the above-specified application fields because of their complexity [8]. Because of its benefits, which include ease of implementation, flexibility, and the ability to avoid becoming stuck in local optima, the metaheuristics algorithm (MA) is able to identify the best or nearly optimal solution [9].

Constrained optimization problems (COPs) are a significant topic in the optimization community. Compared to unconstrained optimization issues, limited optimization problems are more challenging to solve [10]. Consequently, there are substantial theoretical and practical advantages to studying solution techniques for limited optimization problems. Finding choice variables that may maximize or minimize the objective function while meeting linear and nonlinear restrictions inside the search space is a crucial step in addressing restricted optimization problems [11,12]. Constrained optimization issues have been solved using a variety of linear and nonlinear numerical optimization approaches throughout the last ten years. Constrained optimization issues can be efficiently solved using these numerical optimization strategies. Nevertheless, gradient information is necessary for the majority of these numerical optimization techniques [13,14]. For many real-world optimization situations, obtaining the gradient information is difficult. Consequently, many complicated restricted optimization problems are hard to solve using numerical optimization algorithms [15]. In contrast to numerical optimization algorithms, swarm intelligence optimization algorithms exhibit strong flexibility and accuracy in solving constrained optimization problems. Consequently, an increasing number of swarm intelligence algorithms are being used to solve constrained optimization problems [16].

Hashim et al. [17] proposed a novel metaheuristic group method named snake optimization (SO). The iterative process of the SO algorithm offers more optimization techniques and fewer adjustment parameters. Nevertheless, the SO algorithm suffers from a slow convergence speed in the early stage, poor stage interaction, high stochasticity in the initial population, and a tendency to converge quickly to a local optimal solution. In view of the current problems of the SO algorithm, this study examines the improvement of the optimization method proposed by previous researchers. Although this scheme improves the efficiency of algorithm optimization, the optimization speed is relatively slow in the early stage of optimization iteration. To enhance the algorithm’s optimization ability, the refined methods in [18,19] utilized multi-strategy boosting to increase the diversity of particles in the final iteration step. Although this method has achieved certain results, the improved snake optimization algorithm has a slow convergence speed and the optimization effect is not significant enough. Ruba et al. introduced three evolutionary crossover operators (i.e., one-point crossover, two-point crossover, and uniform crossover) to improve the search space exploration of the BSO algorithm and controlled them through switching probabilities. Two newly developed feature selection (FS) algorithms, BSO and BSO-CV, were implemented and evaluated on the real-world COVID-19 dataset and 23 disease benchmark datasets [20]. Although this method improved the accuracy of dataset prediction, the optimization speed was relatively slow in the early stage of optimization iteration. Shourbaji et al. proposed a reptile search algorithm—the snake optimizer (RSA-SO) feature selection algorithm, which uses both the RSA and SO methods in a parallel mechanism. This mechanism reduces the likelihood of the two methods falling into local optima and improves their ability to balance exploration and exploitation [21]. The improved optimization algorithm performs better than the traditional snake optimization algorithm in terms of falling into local optima, but the slow convergence speed of the algorithm is a problem. Belabbes et al. proposed using the snake optimization metaheuristic algorithm to extract parameters for three types of photovoltaic cells: monocrystalline silicon, amorphous silicon, and RTC France. The snake algorithm was applied to single-diode and double-diode models, extracting five and seven parameters, respectively. Root-mean-square-error-based statistical tests were conducted to verify the algorithm’s performance [22]. The optimization effect of the proposed algorithm was not significant enough, and further improvement was needed in terms of algorithm convergence speed, optimization accuracy, and other aspects. To address the issues with the aforementioned algorithms, there is a need for an intelligent optimization algorithm with strong search and optimization capabilities. The snake optimizer (SO) algorithm has the advantages of simple parameter settings and high optimization accuracy, making it suitable for solving complex function optimization problems. However, it also has problems such as a slow convergence speed, susceptibility to local optima, and severe randomness in population initialization.

### 1.2. Contribution

This study applies the optimization of initialization strategy, adaptive phase switching, and population renewal method to further improve the phase linkage and population richness of the SO algorithm. An adaptive *t*-distribution mixed mutation snake optimization method (DTHSO) is suggested based on the study above. The main contribution of this article differs from the work of others in that the following factors:

1. Introduces the Tent chaotic map and quasi-reverse learning strategy to initialize the population, improve the spatial distribution structure of the population’s individual initialization stage, and improve the convergence speed of the algorithm.
2. The exploration and development stages are dynamically chosen based on the algorithm’s fitness throughout the optimization process, which effectively cuts down on needless time spent and quickens the rate of convergence.
3. Swaps out the original algorithm’s population renewal technique with the adaptive *t*-distribution mixed mutation foraging approach. In the development stage, the novel renewal method guarantees population variety; in the exploration stage, it increases the individual richness of the algorithm and boosts optimization capability.
4. Verifies the DTHSO algorithm’s engineering viability through simulation tests on issues in two engineering domains and a comparison with alternative optimization methods on 23 CEC2005 test functions.

## 2. Related Works

Optimization issues with restrictions are tackled using swarm intelligent optimization techniques. Better solution outcomes can be obtained by combining swarm intelligent optimization algorithms with specific constraint-handling strategies, such as the multi-objective optimization method [23], feasibility rule [24], random ranking method [25], and penalty function method [26]. Using the goal function, constraint conditions, and punishment factor as a basis, the adaptive penalty function approach builds a penalty function.

The restricted optimization problem is then converted into a sequence of unconstrained optimization problems by modifying the penalty factor of the penalty function in accordance with the proportion of viable solutions in the current population. In order to tackle the drawbacks of confined optimization issues, a unique adaptive penalty function technique was suggested in [27], which was combined with a straightforward evolutionary approach to create a new evolutionary algorithm for constrained optimization problems. The selection of options from the population that meet the constraints is the foundation of the feasibility rule [28]. Based on each person’s level of constraint violation, the feasibility rule determines which persons move on to the next iteration during the iterative process. When it comes to handling limitations, the feasibility rule offers the benefits of a straightforward theory and simplicity in practice. A unique method for tackling constrained optimization problems, called CSMO, is created by combining the feasibility rule with the improved spider monkey optimization strategy [29]. According to experimental studies, CSMO performs better than the comparison approach when it comes to solving limited optimization issues, which provide a global optimization difficulty. One innovative way to deal with constraints is the random ranking method. Its goal is to rank people according to a likelihood that is based on the objective function value and the degree of constraint violation [30]. Gao et al. developed an estimation distribution method and used the evolutionary sampling technique to produce offspring in order to solve optimization problems with only variable boundary constraints. In constrained optimization, they also suggested a method for resolving any kind of optimization issue with restrictions [31]. By transforming the constraint conditions of a restricted optimization problem into the objective function, the multi-objective constraint-handling approach transforms the constrained optimization problem into a multi-objective unconstrained optimization problem [32]. The multi-objective constraint-handling approach is well adapted to optimization problems in low-feasibility regions and offers excellent convergence performance and impact. Wang et al. proposed a differential multi-objective evolutionary algorithm for multi-mutation strategy fusion [33]. It effectively combined the use of mutation strategies with the multi-objective optimization method in both the early and late stages of iteration, accelerated the algorithm’s convergence, and reduced the high computational cost of the multi-objective optimization method during application. The results showed that the algorithm improved convergence speed and accuracy.

Although significant progress has been made with the above-mentioned methods in handling constrained optimization problems, there is still room for these algorithms to improve in terms of accuracy and efficiency. In 2022, two academicians, Hashim and Hussien, presented the snake optimization method (SO), a novel swarm intelligence optimization method mainly used for solving unconstrained optimization problems. This method is distinguished from other swarm intelligence optimization techniques by its simple concept and small number of parameters. Currently, there is relatively little research on applying the snake optimization technique to solve constrained optimization problems; instead, it is mainly used to tackle unconstrained optimization problems. By combining the snake optimization algorithm with appropriate constraint-handling techniques, we aim to explore a new algorithm with greater solving capabilities and efficiency. This enables the advantages of the snake optimization algorithm in solving unconstrained optimization problems to be applied to solving constrained optimization problems. When dealing with constrained optimization problems, the newly explored method should have a higher solving accuracy and a shorter solving time. However, since the snake optimization process may converge even faster and is prone to being trapped in local optima, this study proposes an adaptive *t*-distribution mixed mutation snake optimization methodology. Initially, the population initialization process of the original method and the quality of the initial solution are enhanced through the use of Tent-based chaotic mapping and the quasi-reverse learning technique. The algorithm’s ability to escape local optima is improved by perturbing and mutating at the optimal solution position to generate new solutions. A mechanism of opposite-sex attraction is proposed to replace the mating strategy in the mating mode of the evolution stage, providing the algorithm with more opportunities for global exploration and development. The improved snake optimization algorithm balances the algorithm’s local and global development while accelerating its convergence speed and improving its accuracy.

## 3. Snake Optimization Algorithm (SO)

Based on the survival strategies of snakes, the SO algorithm is a metaheuristic algorithm. When there is food and a low temperature, snakes will mate; otherwise, they will search for food or consume what is already there [34,35]. The author categorizes these phenomena into many key sections, including population initialization, the split of populations into male and female groups, the assessment of temperature and food amount, and the stages of exploration and development, which are associated with the search process. The ideal solution is reached and the best person in the population is selected after several iterative procedures [36].

### 3.1. Initialize Population

The standard SO algorithm population initialization randomly generates a population in the search space.

(1) $$X_{i}=X_{\min\;}+r\cdot \left(X_{\max\;}-X_{\min\;}\right)$$

The parameter *r* is a random number between [0, 1]. *X_i_* is the initial position of the individual in the *i*-th population. *X*_max_ and *X*_min_ are the upper and lower limits of the search space, respectively.

### 3.2. Division of Male and Female Populations

After completing population initialization, all populations are divided equally into two groups based on the total population, with half being male and the other half being female. The population partitioning formula is

(2) $$N_{m}=N/2$$

(3) $$N_{f}=N-N_{m}$$

where the parameter *N* is the total number of individuals in the population, and *N_m_* and *N_f_* represent the individual numbers of male and female populations, respectively.

### 3.3. Evaluation of Temperature and Food Intake

The definition equation for temperature is

(4) $$\mathrm{Temp}\;=\exp\left(\frac{-t}{T}\right)$$

The parameter *t* is the current number of iterations. *T* is the total number of iterations. As the number of iterations increases, the overall temperature decreases.

The definition equation for food *Q* is:

(5) $$Q=c_{1}\times \exp\left(\frac{t-T}{T}\right)$$

The parameter *c*_1_ is a constant of 0.5. As the number of iterations increases, the overall amount of food increases [37].

### 3.4. Exploration Stage

When the food quantity *Q* < 0.25, the snake searches for food by selecting a random position and then updates its position. The position update formula at this stage is as follows:

(6) $$X_{i,m}(t+1)=X_{\mathrm{rand},m}(t)\pm c_{2}\times A_{m}\times \left(\left(X_{\max\;}-X_{\min\;}\right)\times \mathrm{rand}+X_{\min\;}\right)$$

(7) $$X_{i,f}(t+1)=X_{\mathrm{rand},f}(t)\pm c_{2}\times A_{f}\times \left(\left(X_{\max\;}-X_{\min\;}\right)\times \;\mathrm{rand}\;+X_{\min\;}\right)$$

$X_{i,m}$ and $X_{i,f}$ respectively represent the positions of the male and female snakes. $X_{\mathrm{rand},m}$ and $X_{\mathrm{rand},f}$ represent the positions of randomly selected male and female snakes, respectively. The parameter *rand* is a random number between [0, 1]. The parameters and are the ability of male and female snakes to search for food, respectively. The calculation method is as follows:

(8) $$A_{m}=\exp\left(\frac{-f_{\mathrm{rand},m}}{f_{i,m}}\right)$$

(9) $$A_{f}=\exp\left(\frac{-f_{\mathrm{rand},f}}{f_{i,f}}\right)$$

The parameter $f_{\mathrm{rand},m}$ represents the fitness of $X_{\mathrm{rand},m}$; $f_{i,m}$ represents the fitness of $X_{i,m}$; $f_{rand,f}$ represents the fitness of $X_{\mathrm{rand},f}$; and $f_{i,f}$ represents the fitness of $X_{i,f}$. Fitness is a metric used in algorithms to measure the quality of a solution, calculated through a fitness function to guide the algorithm in searching for better solutions. The parameter $c_{2}$ is a constant of 0.05 [38].

### 3.5. Development Phase

When the amount of food is Q>0.25 and Temp≥0.6, the ambient temperature is in a hot state, and the snake only searches for food. The formula for updating its position is

(10) $$X_{i,j}(t+1)=X_{\mathrm{food}\;}\pm c_{3}\times \;\mathrm{Temp}\;\times \;\mathrm{rand}\;\times \left(X_{\mathrm{food}\;}-X_{i,j}(t)\right)$$

The parameter $X_{i,j}$ is the position of the snake. $X_{\mathrm{food}\;}$ is the current optimal individual position. The parameter $c_{3}$ is a constant of 2.

When Q>0.25 and Temp<0.6 are met, the ambient temperature is in a cold state, and the snake will be in a combat or mating mode.

(1) Combat mode.

Update the position in combat mode to

(11) $$X_{i,m}(t+1)=X_{i,m}(t)+c_{3}\times FM\times \;\mathrm{rand}\;\times \left(Q\times X_{\mathrm{best},f}-X_{i,m}(t)\right)$$

(12) $$X_{i,f}(t+1)=X_{i,f}(t)+c_{3}\times FF\times \;\mathrm{rand}\;\times \left(Q\times X_{\mathrm{best},m}-X_{i,f}(t)\right)$$

$X_{\mathrm{best},f}$ and $X_{\mathrm{best},m}$ represent the optimal individual positions in the male and female populations, respectively. The parameters *FM* and *FF* represent the combat capabilities of male and female snakes, respectively. The parameters *FM* and *FF* are calculated by the following formula:

(13) $$FM=\exp\left(\frac{-f_{\mathrm{best},f}}{f_{i}}\right)$$

(14) $$FF=\exp\left(\frac{-f_{\mathrm{best},m}}{f_{i}}\right)$$

The parameters $f_{\mathrm{best},f}$ and $f_{\mathrm{best},m}$ are the optimal fitness for female and male snakes, respectively. The parameter $f_{i}$ is the fitness of an individual *i*.

(2) Mating mode.

Update the position during the mating mode to

(15) $$X_{i,m}(t+1)=X_{i,m}(t)+c_{3}\times M_{m}\times \;\mathrm{rand}\;\times \left(Q\times X_{i,f}(t)-X_{i,m}(t)\right)$$

(16) $$X_{i,f}(t+1)=X_{i,f}(t)+c_{3}\times M_{f}\times \;\mathrm{rand}\;\times \left(Q\times X_{i,m}(t)-X_{i,f}(t)\right)$$

The parameters $M_{m}$ and $M_{f}$ represent the mating ability of male and female snakes, respectively. The calculation formula is

(17) $$M_{m}=\exp\left(\frac{-f_{i,f}}{f_{i,m}}\right)$$

(18) $$M_{f}=\exp\left(\frac{-f_{i,m}}{f_{i,f}}\right)$$

The parameters $f_{i,m}$ and $f_{i,f}$ represent the fitness of the *i*-th male and female individuals, respectively. If the snake egg is hatched, the worst male and female individuals in the population are randomly replaced.

(19) $$X_{\mathrm{worst},m}=X_{\min}+\mathrm{rand}\times \left(X_{\max}-X_{\min\;}\right)$$

(20) $$X_{\mathrm{wors},f}=X_{\min}+\mathrm{rand}\times \left(X_{\max}-X_{\min}\right)$$

The parameters $X_{\mathrm{worst},m}$ and $X_{\mathrm{wors},f}$ represent the worst individuals in the male and female snake populations, respectively [39].

## 4. Snake Optimization Algorithm with Adaptive *t*-Distribution Mixed Mutation

The SO algorithm is an algorithm with a strong optimization ability, but it is easy to fall into the local optimal solution in the local development stage, that is, its global optimization search ability is insufficient. Therefore, in order to better balance the global exploration ability and local development ability of the algorithm, accelerate the convergence speed of the algorithm, and enhance its robustness, improvement strategies are proposed.

### 4.1. Chaotic Map Based on Tent and Quasi-Reverse Learning Strategy

The diversity of the initial location of the population plays an important role in the snake optimization algorithm to obtain the global optimal solution. Chaotic motion has the characteristics of randomness, ergodicity, regularity, and sensitivity to the initial value, which help to enhance intelligent algorithms to jump out of local optimal solutions and obtain better global optimization search capabilities [40]. In chaotic maps, the random numbers generated by Tent chaotic maps between [0, 1] are relatively uniform, which can better enhance the diversity of the initial population positions of snake optimization algorithms. Therefore, the Tent chaotic map is introduced to initialize the snake position [41].

The chaotic sequence based on Tent chaotic mapping is

(21) $$z_{i+1}=\left\{\begin{array}{ll} \frac{z_{i}}{\varepsilon }, & 0\leq z_{i}\leq \varepsilon \\ \frac{1-z_{i}}{1-\varepsilon }, & \varepsilon <z_{i}\leq 1 \end{array}\;(i=1,2,\cdots )\right.$$

The parameter $z_{i}$ is the *i*-th chaotic value of the chaotic sequence, $z_{i}\in [0,1]$. The parameter ε is the control parameter, and in this article, ε is taken as 0.6.

According to Equation (21), the initial position of individual snake groups based on *Tent* chaotic mapping can be obtained with

(22) $$X_{i}=b_{0}+z_{i}\left(b_{1}-b_{0}\right)\;\left(i=1,2,\cdots ,N_{p}\right)$$

In order to further improve the diversity of the initial positions of snake groups, quasi-reverse learning strategy 1 is introduced. Based on the basic principle of the quasi-reverse learning strategy, if there exists a point *x* ($x\in [c_{1},c_{2}]$, where *c*_1_ and *c*_2_ are the minimum and maximum values of the *x* value interval, respectively), then its quasi-reverse point $x^{*}$ is defined as

(23) $$x^{*}=\frac{c_{1}+c_{2}}{2}+\left(\frac{c_{1}+c_{2}}{2}-x\right)r_{d}$$

According to Formula (22), $x^{*}$ is a uniformly distributed random number within the interval $\left[(c_{1}+c_{2})/2,(c_{1}+c_{2})-x\right]$. Combining *Tent* chaotic mapping with quasi-reverse learning strategy forms the *Tent* chaotic mapping quasi-reverse learning strategy. Using Equations (22) and (23), the initial position of snake swarm individuals based on Tent chaotic mapping quasi-reverse learning strategy is obtained.

### 4.2. Adaptive t-Distribution Mutation SO Algorithm Strategy (DTHSO)

The introduction of Cauchy mutation and Gaussian mutation into intelligent optimization algorithms has been proven to be effective in improving algorithm performance. Among them, Cauchy mutation can enrich the population diversity, while Gaussian mutation can make the algorithm obtain a good local search ability. Cauchy distribution and Gaussian distribution are two special forms of t-distribution [42]. With the increase in the number of iterations and the degree of the freedom parameter *t*, the curve of *t*-distribution gradually approaches Gaussian distribution, changing from Cauchy distribution to Gaussian distribution [43].

Generating a new solution that conforms to the mutation of the *t*-distribution near the position of the optimal solution can combine the advantages of Gaussian distribution and Cauchy distribution at the same time. In the early stage of algorithm iteration, the value of the degree of freedom parameter *t* is small [44]. At this time, the *t*-distribution mainly presents the characteristics of the Cauchy distribution, enriches the diversity of the population, and effectively improves the global search ability of the algorithm. At the middle and late stage of the iteration, the value of the degree of freedom parameter *t* is large, and the *t*-distribution is infinitely close to the Gaussian distribution, which enhances the local development ability of the algorithm and improves its convergence accuracy. In order to enrich the population diversity in the early stage and retain the elite solution of the seagull population in the later stage, adaptive parameters are introduced at the same time. In the early stage of iteration, a larger value can be taken, and the new solution generated by *t*-distribution variation can be used to increase population diversity [45]. With the increase in the number of iterations, the algorithm gradually approaches the optimal solution, and the influence of the adaptive parameter control *t*-distribution on the new solution gradually decreases, fully retaining the elite solution of the seagull population. The expressions for the sum of the new solutions conforming to the variation in the *t*-distribution are shown in Formulas (24) and (25).

(24) $$P_{\mathrm{news}\;}^{t}=P_{bs}^{t}+\omega \cdot TD(t)\cdot P_{bs}^{t}$$

(25) $$\omega =a+(b-a)\cdot \frac{T-t}{T}$$

In the formulas, *TD*(*t*) represents the *t*-distribution of the degree of freedom parameter *t*, a=0.1,b=1, and *T* is the maximum number of iterations.

According to a certain probability, a new solution is accepted with an adaptive *t*-distribution variation and the parameter pe∈[0,1] can be randomly generated. The determination of the new optimal snake individual position is shown in Formula (26).

(26) $$P_{\mathrm{best}\;}=\left\{\begin{array}{cc} P_{bs} & pe>0.5\\ P_{news\;} & pe⩽0.5 \end{array}\right.$$

In this way, when the algorithm performs iterative optimization, there will be two options when determining the optimal solution through probability: one is to continue to select the optimal solution according to the original algorithm, maintaining population diversity while retaining the elite solution; the second is to choose a new solution generated by the mutation perturbation of the adaptive t-distribution, which combines the advantages of Gaussian distribution and Cauchy distribution.

### 4.3. Heterogeneous Attraction Strategy

When the standard SO algorithm is in mating mode, its location update depends on the position and fitness of the *i*-th male and female snakes, and there is a certain probability that the worst individual in the two populations will be replaced. Although all individuals will be closer to the optimal coverage in the whole process, they are also more likely to fall into local optima and cannot jump out. In order to achieve this goal, an opposite attraction strategy is proposed to replace the mating mode [46].

Firstly, the three optimal individuals in the current male and female snake populations are reserved to the next generation, and then the moved positions are calculated according to the three optimal individuals of the opposite sex. Here, the positional changes in male snakes are explained, and the same is true for female snakes. Its calculation formula is

(27) $$X_{m,1}(t)=X_{\mathrm{one},f}(t)-A_{1}\times D_{\mathrm{one},f}$$

(28) $$X_{m,2}(t)=X_{\mathrm{two},f}(t)-A_{2}\times D_{\mathrm{two},f}$$

(29) $$X_{m,3}(t)=X_{\mathrm{three},f}(t)-A_{3}\times D_{\mathrm{three},f}$$

(30) $$X_{m}(t+1)=\frac{X_{m,1}+X_{m,2}+X_{m,3}}{3}$$

$X_{m,1}(t)$, $X_{m,2}(t)$, and $X_{m,3}(t)$ represent, respectively, the positions of the third-generation male snake after being attracted by the current optimal three female individuals. $X_{\mathrm{one},f}(t)$, $X_{\mathrm{two},f}(t)$, and $X_{\mathrm{three},f}(t)$ represent, respectively, the optimal three population positions for the third-generation female snake. Parameter *A* is the convergence factor, and its calculation formula is Equation (31). $D_{\mathrm{one},f}$, $D_{\mathrm{two},f}$, and $D_{\mathrm{three}\;,f}$ are the positional parameters of the three populations with the best reference distance for the female snake, and their calculation formulas are Equations (30)–(32).

(31) $$A=2\times (\mathrm{rand}-1)\times \left(1-\frac{t}{T}\right)$$

Among them, the parameter *t* is the current number of iterations, and *T* is the total number of iterations.

(32) $$D_{\mathrm{one},f}=\left|C_{1}\times X_{\mathrm{one},f}-X_{m}(t)\right|$$

(33) $$D_{\mathrm{two},f}=\left|C_{2}\times X_{\mathrm{two},f}-X_{m}(t)\right|$$

(34) $$D_{\mathrm{three},f}=\left|C_{3}\times X_{\mathrm{three},f}-X_{m}(t)\right|$$

Among them, the parameter *C* is the oscillation factor.C=2×rand.

When *Q* > 0.25 and Temp < 0.6, the execution probabilities of the standard SO algorithm in combat mode and mating mode are 0.6 and 0.4, respectively. In several experiments, it is found that improving the execution probability of the opposite attraction strategy can further improve the optimization ability of the algorithm. While the algorithm is in combat mode, coverage undergoes significant changes as the iteration progresses. This is because the battle mode population position update adopts a larger step size, so that it can quickly find better coverage. However, once the coverage reaches a certain high level, the algorithm will fall into the local optimal solution and cannot further improve the coverage. When the algorithm is in the phase of the attraction of opposites, except for a few optimal individuals in the population that are completely reserved to the next generation, all other individuals rely on the optimal individuals in the opposites population to update their positions, which can reduce the possibility of falling into local optima. And with the increase in the number of iterations, the optimization step size of the positional update in this stage is also gradually reduced, thus improving the local optimization performance of the algorithm. Therefore, in the DTHSO algorithm, the execution of the combat mode and opposite attraction strategy may be changed to 0.4 and 0.6, so as to improve the optimization ability of the algorithm.

### 4.4. Analysis of Algorithm Time Complexity

Assuming that the time complexity of the SO algorithm is *T*(*n*), the search space dimension is *D*, the population number of the snake optimization algorithm is *N*, and the maximum number of iterations is *T*_max_, then the complexity of population initialization is *O*(*n*) and the complexity of location update in the first stage is *O*(*ND*). In the second stage, the temperature and physical location are calculated first and then the location update is carried out. The complexity is O(ND+1), and the time complexity of the SO algorithm is $T(n)=O[T_{\max}(2ND+1)+N]$. Compared with the SO algorithm, the DTHSO algorithm has three processes added: *Tent* chaotic mapping and quasi-reverse learning, adaptive *t*-distribution mutation, and the anisotropic attraction strategy, and the complexity correspondingly increases. Reverse population initialization generates two different populations using Tent chaotic mapping and reverse learning, with a complexity of *O*(2*n*). The complexity of adaptive *t*-distribution variation is *O*(*D*). The opposite attraction strategy is nested in the second stage position update, and its complexity remains unchanged. Therefore, the time complexity of the DTHSO algorithm is $T(n)=O[2N+T_{\max}(2ND+D+1)]$, with a slight increase in time complexity and little difference.

### 4.5. The Algorithm Process of DTHSO Algorithm

The implementation steps of the DTHSO algorithm are as follows:

Step 1: Based on the objective function that needs to be calculated, establish a mathematical model and determine the optimization objective.

Step 2: Set the number of snake populations and the maximum number of iterations, and initialize the snake population based on the *Tent* chaotic map and quasi-reverse learning strategy based on Formula (19).

Step 3: Divide the population into two groups, male and female, according to Equations (2) and (3); set a fitness function; and calculate the corresponding fitness to find the current best male and female individuals.

Step 4: Define the ambient temperature *Temp* and food quantity *Q* based on Equations (4) and (5).

Step 5: Determine whether to only search for food or engage in battles and mating based on the amount of food *Q*. If *Q* < 0.25, search for food and update the position of the individual snake according to Equation (6).

Step 6: If there is sufficient food and the *Temp* > 0.6, only search for food and eat existing food, and update the position according to Equation (10).

Step 7: Determine whether to enter the combat mode or mating mode based on the random number, rand, of the mode. Update the position of the combat mode according to Equation (11), replace the *FM* in combat mode with Equation (13), replace the best individual with the current individual, and update the position. If the eggs hatch, select the worst individuals and replace them.

Step 8: Process the updated position and update the individual’s historical best value.

Step 9: Update the position again based on the improved adaptive *t*-distribution mutation according to Equation (22).

Step 10: Update individual historical best values using the opposite attraction strategy.

Step 11: Update the global best fit again and update the food location.

Step 12: Determine if the number of iterations has been reached, and if it does not meet the requirements, proceed to the next iteration; if satisfied, end the iteration and output the optimal position.

**Figure 1:**
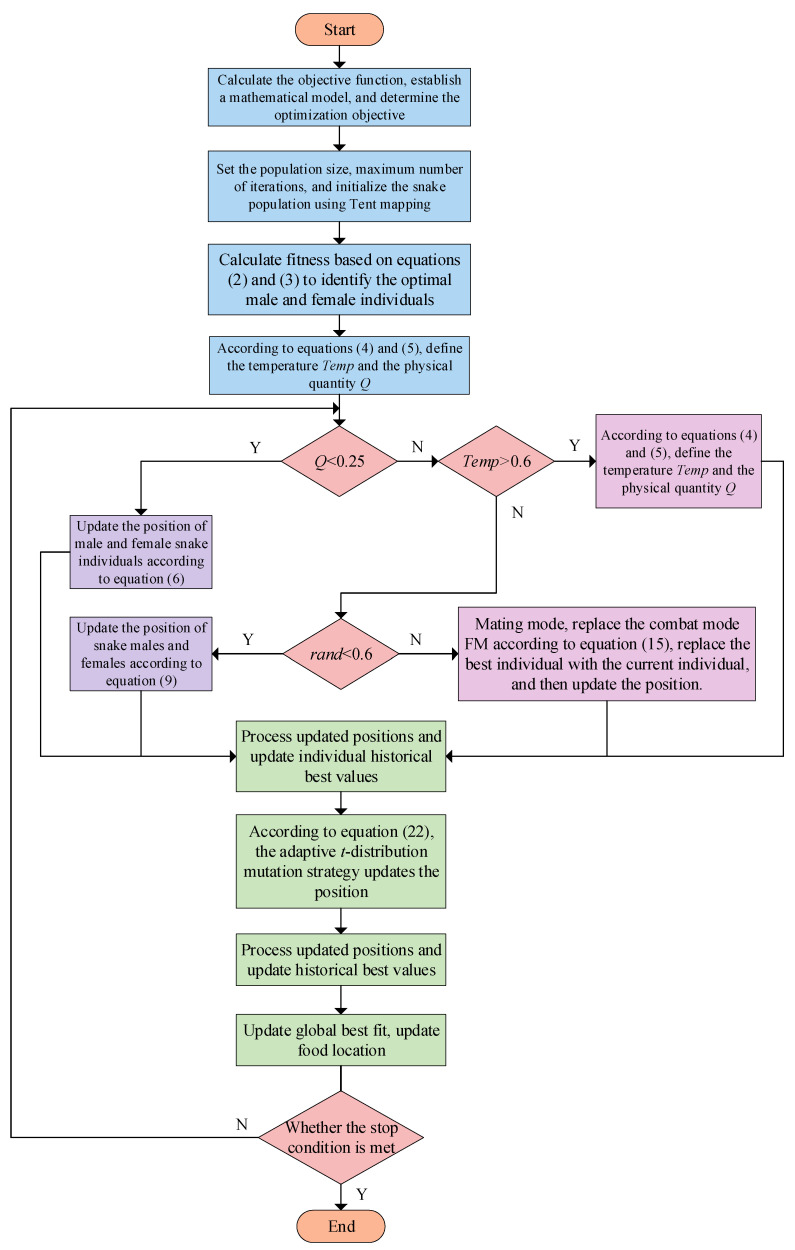
The workflow of the improved DTHSO algorithm.

The workflow of the improved DTHSO algorithm is shown in Figure 1.

## 5. Algorithm Comparison and Result Analysis

### 5.1. Comparison of Test Function Results

The simulation testing environment is configured as follows: the operating system is Windows 10, 64-bit edition; the processor is a 12th Generation Intel Core(TM) i9-12900K (Intel Corporation, Santa Clara, CA, USA) with a base frequency of 3.20 GHz; the system is equipped with 64GB of RAM; and the simulation software utilized is MATLAB R2022b. The algorithms selected for comparison include the Gray Wolf Optimizer (GWO) [47], particle swarm optimization (PSO) [48], Whale Optimization Algorithm (WOA) [49], Dung Beetle Optimizer (DBO) [50], Subtraction-Average-Based Optimizer (SABO) [51], Sand Cat Swarm Optimization (SCSO) [52], standard snake optimization (SO) [17], and the proposed DTHSO algorithm. To ensure fairness in the comparison of each algorithm, the population size for all algorithms is set to 30, and the number of iterations is set to 500. This study employs 23 standard benchmark functions from CEC2005 for comparative experiments, which are designed to mimic varying degrees of difficulty in actual search spaces. Detailed information regarding these functions is presented in Table 1.

**Figure 2:**
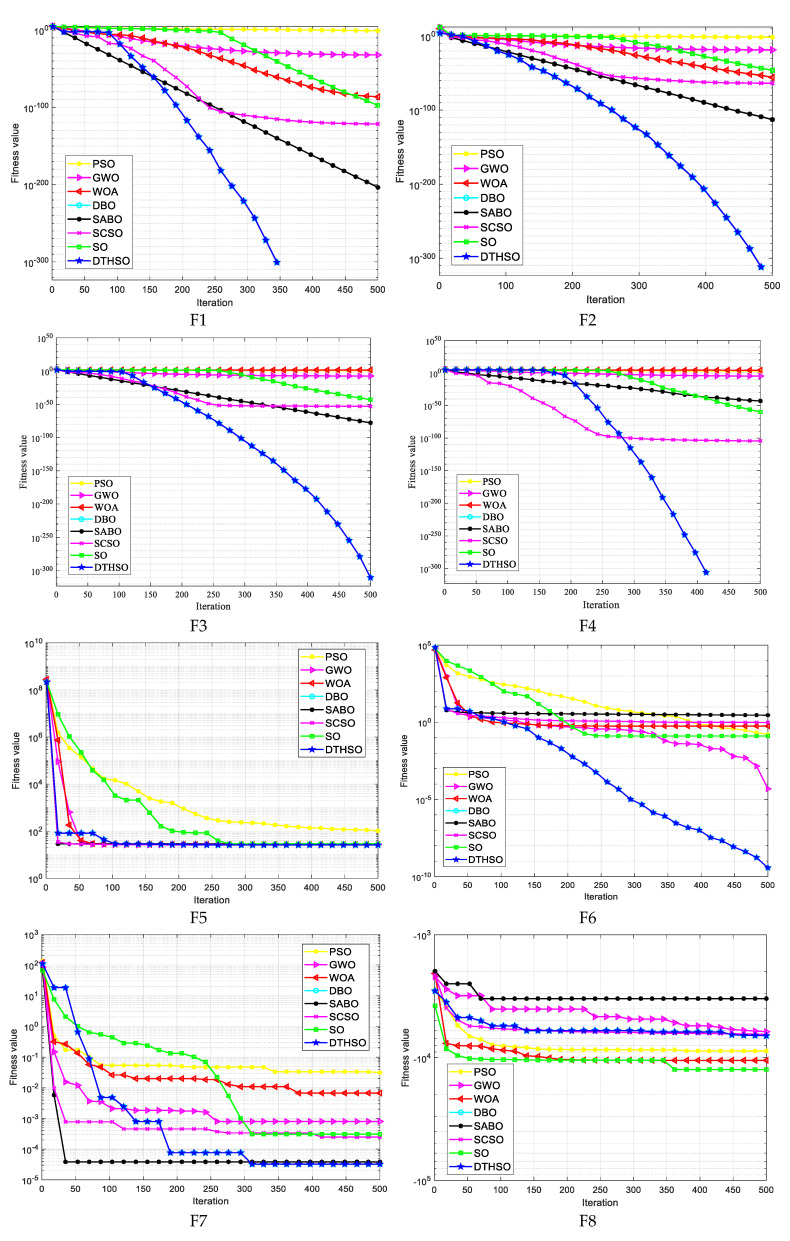

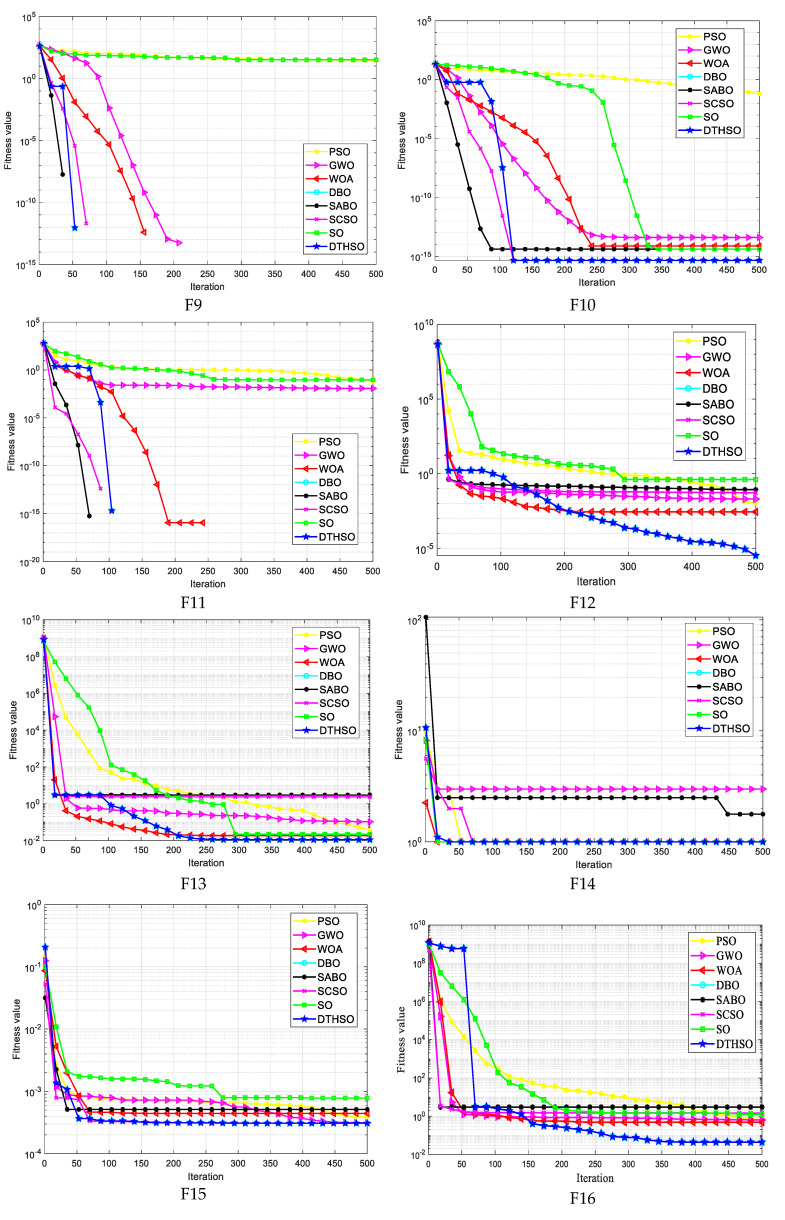

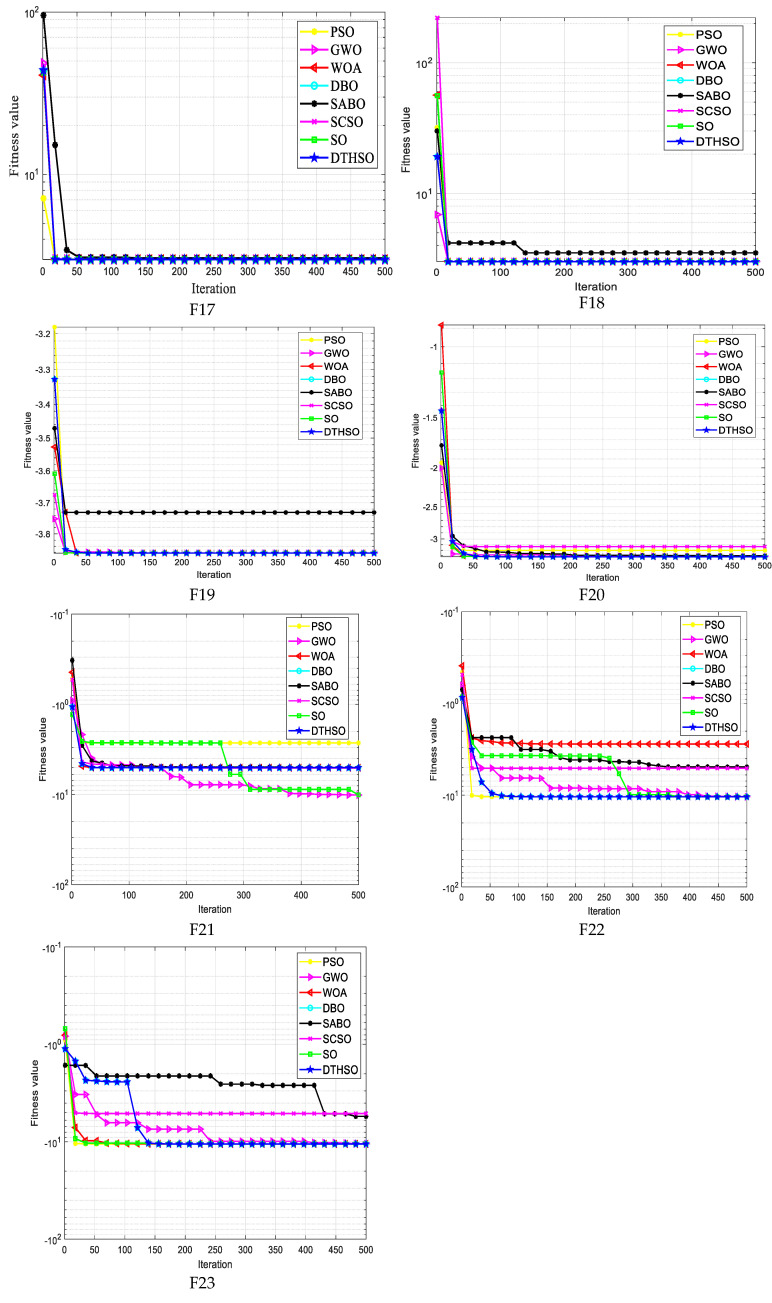
Comparison of iterative computation results for eight algorithms.

**Figure 3:**
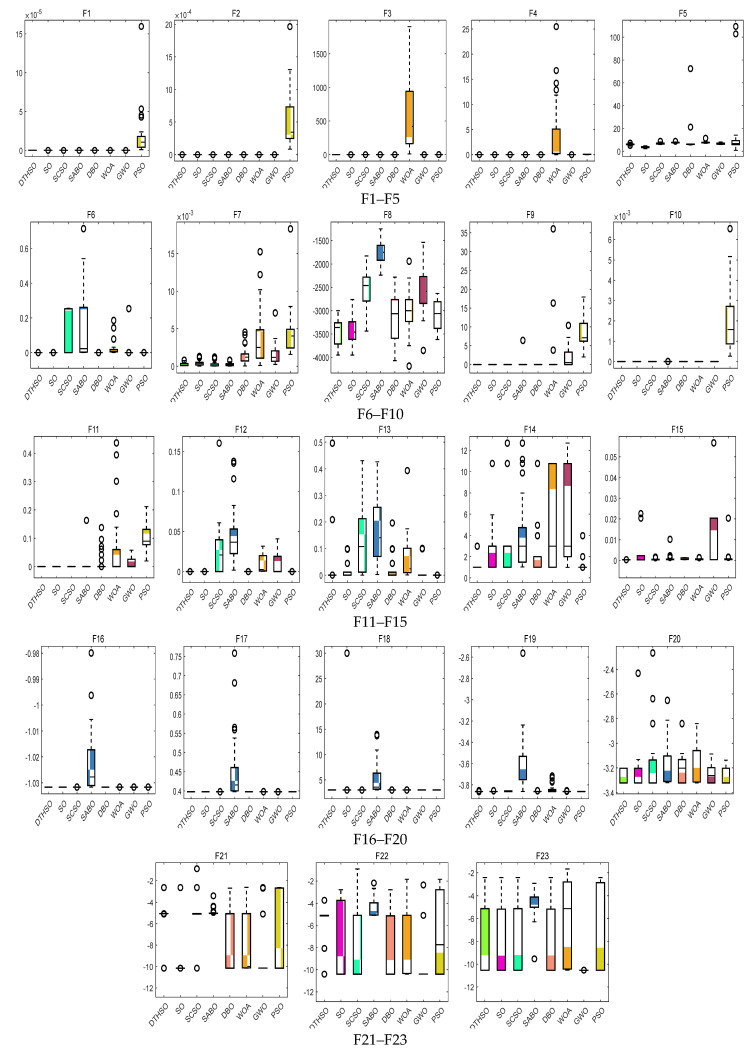
Bar charts of test function results for eight algorithms.

After 30 rounds of average calculation, the iterative calculation results of eight algorithms are shown in Figure 2. The bar charts of the test function results for the eight algorithms are shown in Figure 3. Table 2 displays a comparison of the experimental results for the eight algorithms.

**Figure 4:**
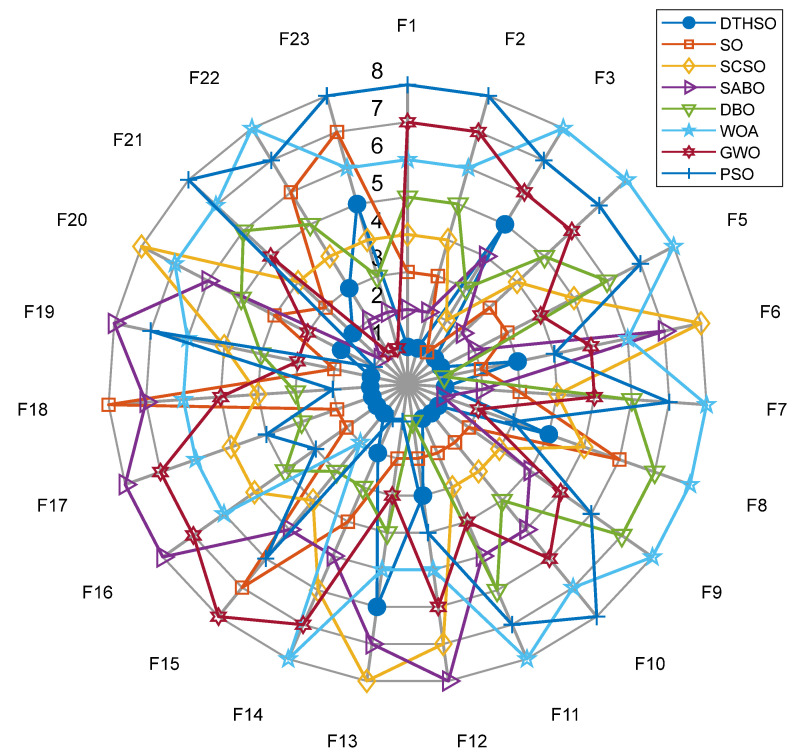
The radar plots of eight algorithms in 23 test functions.

**Figure 5:**
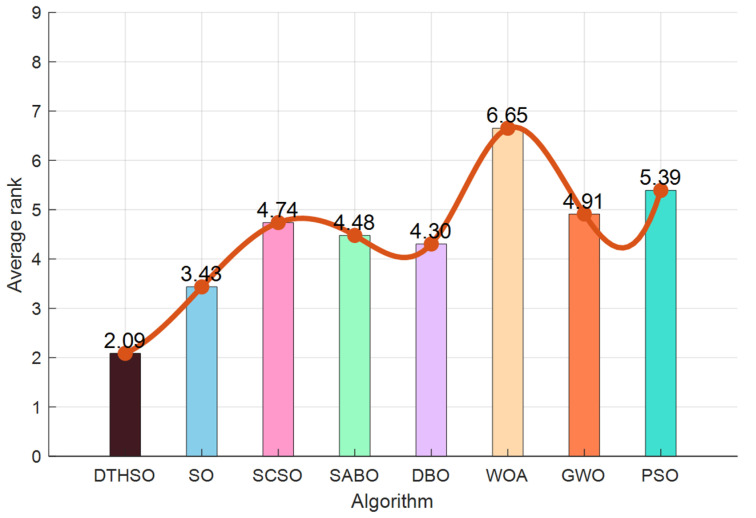
The performance comparison and ranking of the eight additional algorithms.

In order to increase the visual comparability of the data, we added eight algorithms to the radar plots of 23 test functions, as shown in Figure 4. The performance comparison and rankings of the eight additional algorithms are shown in Figure 5.

In order to compare the convergence speed and accuracy of the algorithms more intuitively, this article selects the iterative convergence curves of 23 benchmark test functions for comparison, as shown in Figure 2. Observing the convergence curve of the test function, it can be seen that the improved algorithm DTHSO proposed in this article has a better convergence speed and accuracy than PSO, GWO, WOA, DBO, SABO, SCSO, and the standard SO algorithm. F1~F6 are high-dimensional unimodal functions that reflect the algorithm’s global exploration and convergence capabilities. After using Tent chaotic mapping and the quasi-reverse learning strategy, the initial population can be effectively optimized. Simultaneously adapting the *t*-distribution mutation strategy and the opposite attraction strategy can reduce the search space, help the algorithm to jump out of local optima, and improve the algorithm’s optimization ability. From the convergence curve of the F1–F6 functions, it can be seen that the DTHSO algorithm has a high convergence speed and accuracy, which to some extent indicates that the introduction of the adaptive *t*-distribution mutation strategy and anisotropic attraction strategy has improved the weak global search ability of the SO algorithm. For high-dimensional multimodal functions, they are mainly used to detect the local development ability of the algorithms. From the convergence curve of algorithm comparison, it can be seen that the DTHSO algorithm shows superior performance, which is less likely to fall into local optima compared to other PSO, GWO, WOA, DBO, SABO, SCSO, and standard SO algorithms, improving the shortcomings of the original algorithm. Overall, from the convergence graph, it can be seen that the DTHSO algorithm can also demonstrate significant superiority in the optimization problem of finding the minimum value of a function.

According to the experimental results in Table 2, it can be seen that the DTHSO algorithm proposed in this paper can directly explore the optimal values for F1~F6, and the optimization effect reaches 100%. For F7, the average and the standard deviation of the DTHSO algorithm are also much smaller than those of other algorithms, indicating its high stability. For F7~F10, there is a better verification effect on the algorithm’s global exploration ability. For F11 and F15, the optimization effect of the DTHSO algorithm is relatively ideal. The algorithm proposed in this article is better than PSO, GWO, WOA, DBO, SABO, SCSO, and standard SO algorithms, with a faster convergence speed and the best performance. For functions F16 and F17, PSO, GWO, WOA, DBO, SABO, SCSO, the standard SO algorithm, and the algorithm proposed in this article all have poor performance and are not given here. For F18~F23, the optimization performance of the DTHSO algorithm is not significant compared to other algorithms such as PSO, GWO, WOA, DBO, SABO, SCSO, and the standard SO algorithm, but the average and standard deviation are far better than the other compared algorithms. The above analysis indicates that the overall optimization performance of the DTHSO algorithm is superior to the other seven algorithms.

The radar charts of the 48 algorithms added in the 23 test functions, and the performance comparison and ranking chart of the eight added algorithms are in Figure 5. It can be clearly seen that the DTHSO algorithm proposed in this article has significant advantages and optimal results compared to other algorithms. Compared with the other seven algorithms, it can be seen that our algorithm has significant performance advantages.

In summary, the DTHSO algorithm has significantly improved the optimization performance of 23 benchmark test functions and achieved good stability, effectively improving the shortcomings of the original algorithm. The feasibility and effectiveness of the DTHSO algorithm have been demonstrated. This article proposes a multi strategy improved DTHSO algorithm based on the original SO algorithm, and further improves some behaviors by combining the type of SO and its role in the population. DTHSO is compared with seven other excellent algorithms in 23 commonly used benchmark functions in multiple dimensions, and the convergence curves of each algorithm are analyzed. The significance of the differences is verified by the Wilcoxon sign rank test, and the results fully prove the effectiveness of the improved strategy. The results indicate that the DTHSO algorithm has a good optimization ability and robustness in complex problems.

### 5.2. Comparison of Energy Storage Optimization Scheduling

**Figure 6:**
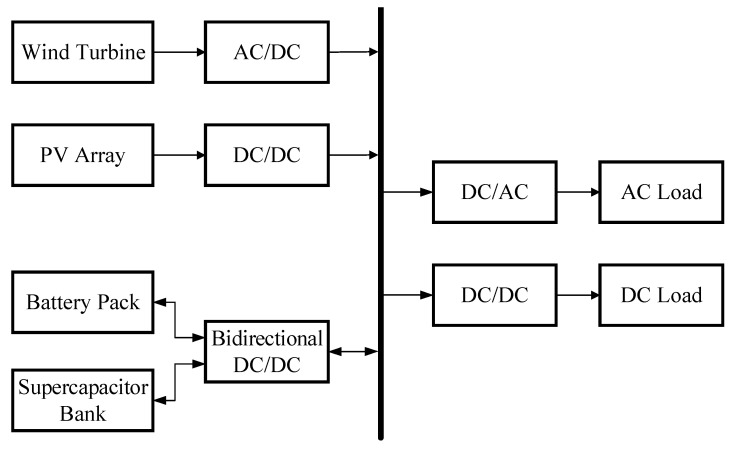
Wind–solar complementary hybrid energy storage structure.

In order to improve the economic efficiency of wind–solar complementary power generation energy storage systems and reduce their operating costs, a capacity optimization configuration model for wind–solar complementary power generation energy storage systems is studied, and the improvement of the particle swarm optimization algorithm and hybrid energy storage capacity optimization method are explored. In the wind–solar complementary power generation system, a battery supercapacitor hybrid is used as an energy storage device. The system composition is shown in Figure 6, which consists of wind turbines, photovoltaic arrays, batteries, supercapacitors, converters, loads, etc.

The life cost cycle (*LCC*), also known as the total lifecycle cost, refers to the sum of all expenses paid during the planning, manufacturing, installation, use, maintenance, and disposal of equipment throughout its lifecycle. To estimate *LCC* reasonably and accurately, a breakdown structure for the entire lifecycle cost is established. Based on the work items of each stage of the entire lifecycle, the cost of the entire lifecycle is gradually decomposed into basic units, forming a unit cost system sorted and arranged in sequence, known as the cost breakdown structure (*CBS*). An estimation model for *LCC* based on the breakdown structure of expenses is established. The cost model, without considering the time value of funds, is a static cost model, where *LCC* is

(35) $$LCC=C_{1}+C_{O}+C_{M}+C_{D}$$

*LCC* is the full lifecycle cost. *C*_1_ is the purchase cost of the equipment. *C_O_* is the operating cost of the equipment. *C_M_* is the maintenance cost of the equipment. *C_D_* represents the processing cost (residual value cost and scrap cost) of the equipment.

The objective function is the full lifecycle cost of hybrid energy storage devices, which can also be defined as the sum of the four major costs. The LCC model of a hybrid energy storage system is

(36) $$\begin{array}{l} \min C=C_{1}+C_{O}+C_{M}+C_{D}\\ \;\;\;=(1+f_{ob}+f_{mb}+f_{db})N_{b}P_{b}+(1+f_{oc}+f_{dc})N_{c}P_{c} \end{array}$$

**Figure 7:**
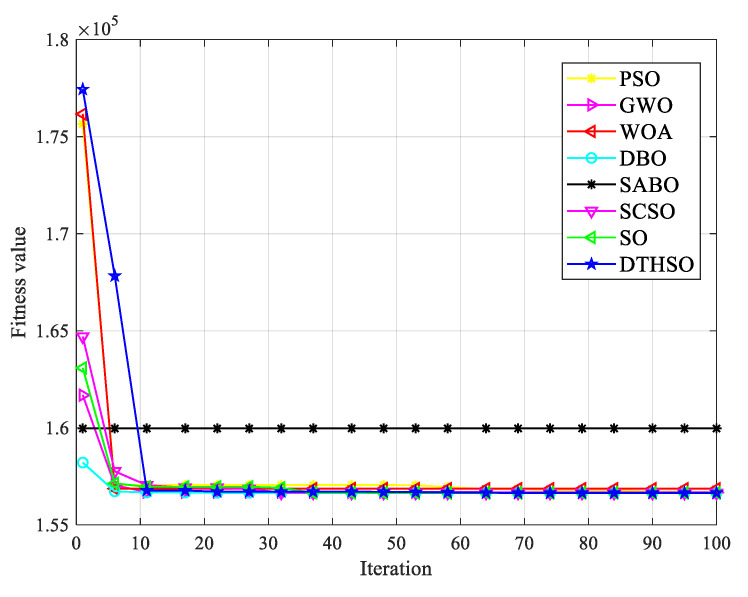
Comparison of the energy storage optimization scheduling of eight algorithms.

**Figure 8:**
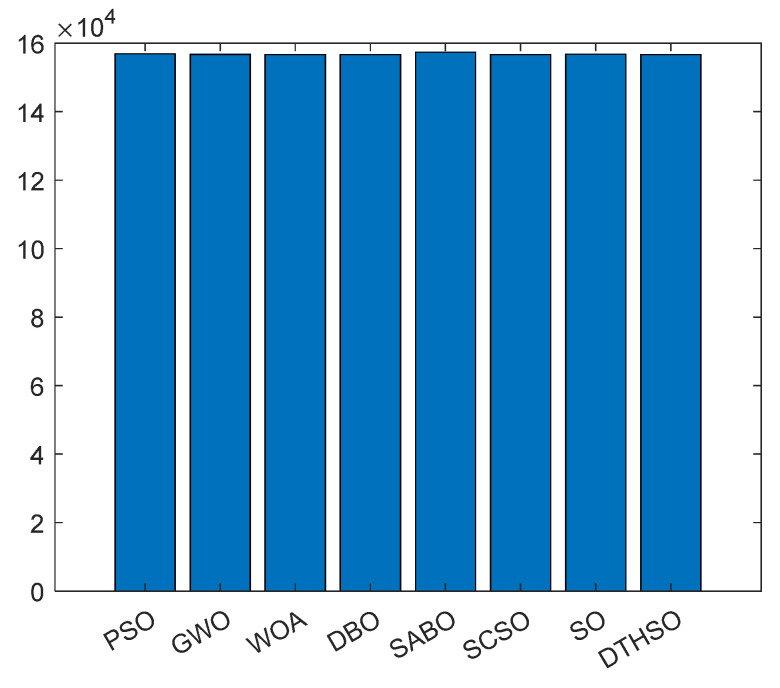
Comparison of minimum lifecycle costs.

**Figure 9:**
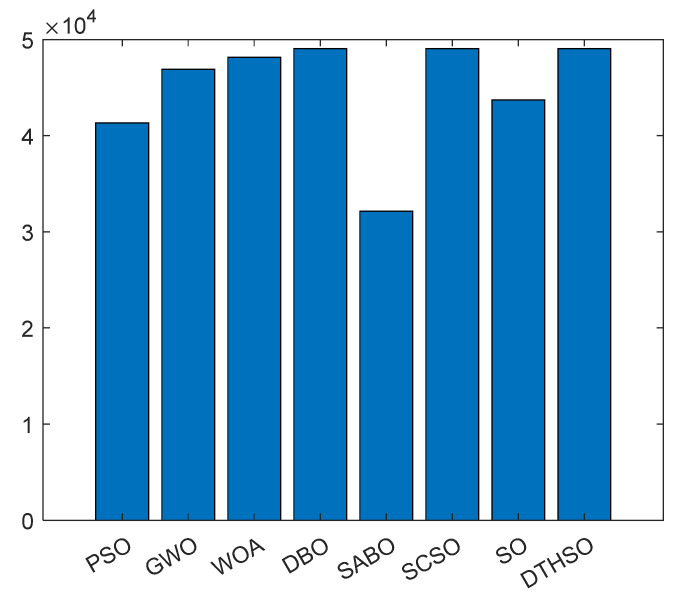
Comparison of battery quantity.

**Figure 10:**
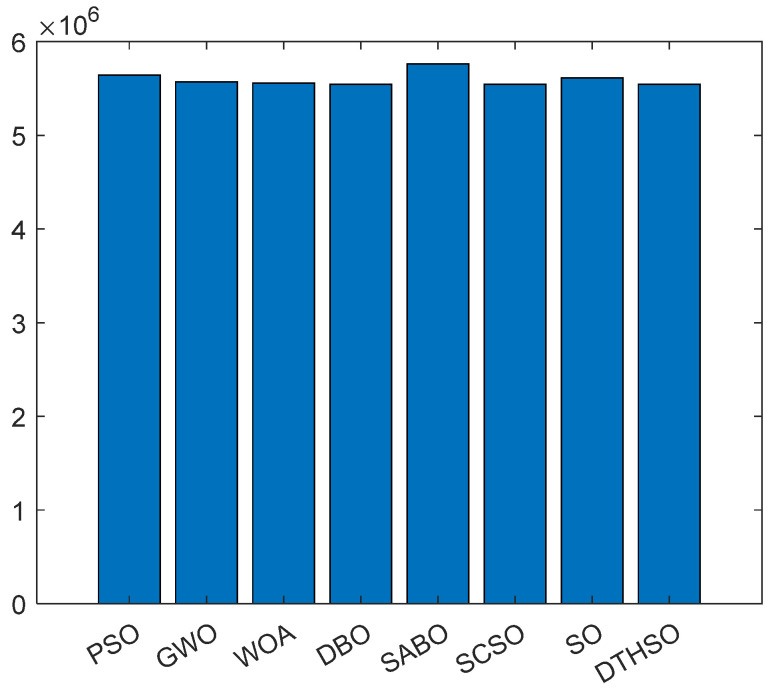
Comparison of the number of capacitors.

The parameters *N_b_* and *N_c_* represent the number of batteries and supercapacitors, respectively. *P_b_* and *P_c_* are the unit prices of batteries and supercapacitors, respectively. *f_ob_* and *f_oc_* are the operating coefficients of the battery and supercapacitor. *f_mb_* and *f_mc_* are the maintenance coefficients for the batteries and supercapacitors. Generally, supercapacitors are maintenance free, so *f_mc_* = 0. *f_db_* and *f_dc_* are the processing coefficients for the batteries and supercapacitors. The specific mathematical model can be found in reference [53,54]. The comparison of capacity optimization iterations for the hybrid energy storage systems using eight algorithms is shown in Figure 7. The minimum lifecycle cost of the intelligent optimization algorithm is shown in Figure 8. The number of batteries is shown in Figure 9. The number of capacitors is shown in Figure 10.

From the simulation results, it can be seen that when using PSO, GWO, WOA, DBO, SABO, SCSO, and standard SO algorithms, the convergence speed is relatively slow, requiring approximately 15 iterations to find the approximate optimal solution. The DTHSO algorithm proposed in this article shows a significant acceleration in convergence speed, with convergence occurring after approximately 10 times. The results obtained using PSO, GWO, WOA, DBO, SABO, SCSO, and standard SO algorithm methods all meet the requirements of the minimum load outage rate. The optimization effect of the DTHSO algorithm proposed in this paper is superior to the other seven intelligent optimization algorithms.

Compared to PSO, GWO, WOA, DBO, SABO, SCSO, and standard SO algorithms, it can be seen that using the DTHSO algorithm requires 49,827 batteries and 5,623,400 supercapacitors. At this time, the minimum lifecycle cost is about CNY 159,880, and the load outage rate is 0.032. When using an asymmetric acceleration factor, approximately 47,212 batteries and 5,685,300 supercapacitors need to be configured, resulting in a minimum lifecycle cost of CNY 157,130 and a load shortage rate of 0.035. It can be seen that the DTHSO algorithm proposed in this article has a better optimization ability. The capacity configuration optimization design of hybrid energy storage devices for batteries and supercapacitors is carried out with the goal of minimizing the total lifecycle cost of the system and the corresponding constraints, such as power outage rate. This includes the design of a capacity configuration optimization model and the use of the DTHSO algorithm proposed in this paper. Simulation results show that when using the DTHSO algorithm proposed in this paper, it converges approximately 10 times, accelerating the convergence speed and having a better optimization ability. This optimization model using the DTHSO algorithm has certain referential value for the capacity configuration and the economy of hybrid energy storage devices in wind–solar complementary power generation systems.

## 6. Conclusions

To overcome the shortcomings of traditional snake optimization processes, including poor population diversity, sluggish convergence, and inadequate interaction during the exploration and exploitation stages, this work suggests an adaptive *t*-distribution mixed mutation snake optimization approach. This method starts with a quasi-reverse learning strategy and Tent chaotic mapping. It employs an opposite-sex attraction approach in place of the mating strategy for optimization and substitutes an adaptive *t*-distribution mixed mutation foraging strategy for the original foraging stage technique. Higher solution accuracy and a quicker rate of convergence are provided by the enhanced approach. The DTHSO algorithm shows the highest accuracy among the various approaches, and the outcomes of 23 test functions confirm the optimization strategy’s efficacy. In order to lower the total system lifespan cost and satisfy the related requirements, such as the power outage rate, the capacity configuration optimization design is performed for hybrid energy storage devices, such as batteries and supercapacitors. The number of batteries needed for configuration is significantly reduced when an optimization model for capacity configuration is created and an adaptive *t*-distribution mixed mutation in a snake-shaped optimization approach is used. In wind–solar complementary power generating systems, this has a predetermined reference value for the economy and for the capacity arrangement of hybrid energy storage devices.

The advanced SO algorithm demonstrates superior optimization abilities, resilience, and practicality. Nevertheless, when it comes to the function testing and capacity optimization of hybrid energy storage systems, it has not consistently yielded uniform outcomes. There remain certain constraints. The primary challenge is that for two or three test functions, the optimization results of the DTHSO technique are less favorable than those of the traditional SO method. Even though it can circumvent local optima in the majority of situations, the DTHSO algorithm sometimes fails to break free from local optima. In comparison with previous swarm intelligence algorithms, the DTHSO approach substantially enhances the performance for unimodal functions, yet has a relatively more limited influence on some multimodal functions. In light of these concerns, relevant improvement techniques will be put forward and implemented to address real-life problems in diverse fields, such as signal processing, image segmentation, and unmanned aerial vehicle (UAV) route optimization.

## Figures and Tables

**Table 1:** CEC2005 test functions.

Function	Equation	Dimension	Bounds	Optimum
F1	$\sum_{i=1}^{d}x_{i}^{2}$	30	[−100, 100]	0
F2	$\sum_{i=1}^{d}\left|x_{i}\right|+\prod_{i=1}^{d}\left|x_{i}\right|$	30	[−10, 100]	0
F3	${\sum_{i=1}^{d}\left(\sum_{j=1}^{i}x_{j}\right)}^{2}$	30	[−100, 100]	0
F4	$\max\left\{\left|x_{i}\right|,1\leq i\leq d\right\}$	30	[−100, 100]	0
F5	$\sum_{i=1}^{d-1}\left[100{\left(x_{i+1}-x_{i}^{2}\right)}^{2}+{\left(x_{i}-1\right)}^{2}\right]$	30	[−10, 100]	0
F6	$\sum_{i=1}^{d}{\left(\left|x_{i}+0.5\right|\right)}^{2}$	30	[−100, 100]	0
F7	$\sum_{i=1}^{d}ix_{i}^{4}+rand(0,1)$	30	[−1.28, 1.28]	0
F8	$\sum_{i=1}^{d}\left|x_{i}\sin(x_{i})+0.1x_{i}\right|$	30	[−10, 100]	0
F9	$10d+\sum_{i=1}^{d}\left[x_{i}^{2}-10\cos(2\pi x_{i})\right]$	30	[−100, 100]	0
F10	$-20\exp\left(-0.2\sqrt{\frac{1}{d}\sum_{i=1}^{d}x^{2}}\right)-\exp\left(\frac{1}{d}\sum_{i=1}^{d}\cos(2\pi x_{i})\right)+20+\exp(1)$	30	[−5.12, 5.12]	0
F11	$\frac{1}{4000}\sum_{i=1}^{d}x_{i}^{2}-\prod_{i=1}^{d}\cos\frac{x_{i}}{\sqrt{i}}+1$	30	[−32, 32]	0
F12	$\begin{array}{l} F12=\frac{\pi }{d}\left\{10\sin(\pi y_{1})+\sum_{i=1}^{d-1}{\left(y_{i}-1\right)}^{2}[1+10\sin^{2}(\pi y_{i+1})]+{\left(y_{n}-1\right)}^{2}\right\}\\ \;\;\;+\sum_{i=1}^{d}u(x_{i},10,100,4)\\ y_{i}=1+\frac{x_{i}+1}{4}u\left(x_{i},a,k,m\right)=\left\{\begin{array}{c} k{\left(x_{i}-a\right)}^{m},x_{i}>a\\ 0,-a<x_{i}<a\\ k{\left(-x_{i}-a\right)}^{m},x_{i}<-a \end{array}\right. \end{array}$	30	[−600, 600]	0
F13	$0.1\left\{\sin^{2}(3\pi x_{1})+\sum_{i=1}^{d-1}{\left(x_{i}-1\right)}^{2}[1+\sin^{2}(3\pi x_{i}+1)]+{\left(x_{n}-1\right)}^{2}[1+\sin^{2}(2\pi x_{n})]\right\}+\sum_{i=1}^{d}u(x_{i},5,100,4)$	30	[−50, 50]	0
F14	${\left(\frac{1}{500}+\sum_{j=1}^{25}\frac{1}{j+\sum_{i=1}^{2}{\left(x_{i}-a_{ij}\right)}^{6}}\right)}^{-1}$	2	[−65, 65]	0
F15	$\sum_{i=1}^{11}{\left[a_{i}-\frac{x_{i}(b_{i}^{2}+b_{i}x_{2})}{b_{i}^{2}+b_{i}x_{3}+x_{4}}\right]}^{2}$	4	[−5, 5]	0.0003
F16	$4x_{1}^{2}-2.1x_{1}^{4}+\frac{1}{3}x_{1}^{6}+x_{1}x_{2}-4x_{2}^{2}+4x_{2}^{4}$	2	[−5, 5]	−1.0316
F17	${\left(x_{2}-\frac{5.1}{4\pi^{2}}x_{1}^{2}+\frac{5}{\pi }x_{1}-6\right)}^{2}+10(1-\frac{1}{8\pi })\cos x_{1}+10$	2	[−5, 10]	0.3978
F18	$\begin{array}{l} {}[1+{\left(x_{1}+x_{2}+1\right)}^{2}(19-14x_{1}+3x_{1}^{2}-14x_{2}+\\ 6x_{1}x_{2}+3x_{2}^{2})]\times [30+{\left(2x_{1}-3x_{2}\right)}^{2}\times (18-32x_{1}\\ +12x_{1}^{2}+48x_{2}-36x_{1}x_{2}+27x_{2}^{2})] \end{array}$	2	[−2, 2]	3
F19	$-\sum_{i=1}^{4}c_{i}\exp\left(-\sum_{j=1}^{3}a_{ij}{\left(x_{j}-p_{ij}\right)}^{2}\right)$	3	[1, 3]	−3.86
F20	$-\sum_{i=1}^{4}c_{i}\exp\left(-\sum_{j=1}^{6}a_{ij}{\left(x_{j}-p_{ij}\right)}^{2}\right)$	6	[0, 1]	−3.32
F21	$-\sum_{i=1}^{5}{\left[(X-a_{i}){\left(X-a_{i}\right)}^{T}+c_{i}\right]}^{-1}$	4	[0, 10]	−10.1532
F22	$-\sum_{i=1}^{7}{\left[(X-a_{i}){\left(X-a_{i}\right)}^{T}+c_{i}\right]}^{-1}$	4	[0, 10]	−10.4028
F23	$-\sum_{i=1}^{10}{\left[(X-a_{i}){\left(X-a_{i}\right)}^{T}+c_{i}\right]}^{-1}$	4	[0, 10]	−10.5363

**Table 2:** Comparison of experimental results of eight algorithms.

Function	Algorithm	Mean	Std	Best	Worst
F1	PSO	7.23×10^−10^	1.29×10^−9^	5.28×10^−12^	5.18 × 10^−9^
	GWO	1.25 × 10^−56^	5.57 × 10^−56^	3.41 × 10^−60^	3.07 × 10^−55^
	WOA	6.99 × 10^−74^	3.83 × 10^−73^	7.73 × 10^−91^	2.1 × 10^−72^
	DBO	1.2 × 10^−102^	6.76 × 10^−102^	3 × 10^−154^	3.7 × 10^−101^
	SABO	1.6 × 10^−199^	0	5.8 × 10^−205^	3.2 × 10^−198^
	SCSO	2.1 × 10^−131^	7.61 × 10^−131^	7.6 × 10^−146^	4.1 × 10^−130^
	SO	3.96 × 10^−161^	1.6 × 10^−160^	2.47 × 10^−174^	8.08 × 10^−160^
	DTHSO	0	0	0	0
F2	PSO	1.45 × 10^−6^	8.83 × 10^−7^	2.94 × 10^−7^	4 × 10^−6^
	GWO	7.94 × 10^−33^	1.55 × 10^−32^	2.9 × 10^−35^	7.8 × 10^−32^
	WOA	4.86 × 10^−51^	2.66 × 10^−50^	3.93 × 10^−60^	1.46 × 10^−49^
	DBO	2.7 × 10^−55^	9.45 × 10^−55^	2.4 × 10^−85^	4.53 × 10^−54^
	SABO	1.5 × 10^−109^	1.9 × 10^−109^	4 × 10^−112^	7.2 × 10^−109^
	SCSO	5.12 × 10^−69^	2.4 × 10^−68^	4.34 × 10^−76^	1.31 × 10^−67^
	SO	2.307 × 10^−87^	5.077 × 10^−87^	5.735 × 10^−91^	2.574 × 10^−86^
	DTHSO	0	0	0	0
F3	PSO	0.002794	0.003631	9.86 × 10^−5^	0.01412
	GWO	1.59 × 10^−24^	4.89 × 10^−24^	5.58 × 10^−31^	2.12 × 10^−23^
	WOA	255.6735	476.0306	0.380031	2629.944
	DBO	3.84 × 10^−89^	2.11 × 10^−88^	8.9 × 10^−161^	1.15 × 10^−87^
	SABO	6.56 × 10^−74^	3.59 × 10^−73^	1.7 × 10^−122^	1.97 × 10^−72^
	SCSO	5.9 × 10^−111^	3.3 × 10^−110^	1 × 10^−126^	1.8 × 10^−109^
	SO	1.96 × 10^−131^	9.46 × 10^−131^	4.85 × 10^−144^	5.19 × 10^−130^
	DTHSO	−2.5 × 10^−27^	4.19 × 10^−26^	0	−7.4 × 10^−26^
F4	PSO	0.001705	0.001292	0.000198	0.00573
	GWO	3.47 × 10^−18^	6.38 × 10^−18^	1.98 × 10^−20^	3.11 × 10^−17^
	WOA	5.260061	13.52821	0.000802	64.69591
	DBO	9.37 × 10^−44^	5.13 × 10^−43^	4.59 × 10^−79^	2.81 × 10^−42^
	SABO	7.44 × 10^−85^	1.47 × 10^−84^	7.91 × 10^−88^	6.65 × 10^−84^
	SCSO	1.77 × 10^−57^	9.08 × 10^−57^	2.16 × 10^−64^	4.98 × 10^−56^
	SO	4.259 × 10^−72^	2.307 × 10^−71^	3.529 × 10^−77^	1.264 × 10^−70^
	DTHSO	0	0	0	0
F5	PSO	8.409876	11.91073	3.796652	69.3338
	GWO	6.497992	0.501592	6.090313	8.062182
	WOA	11.54476	25.59158	6.131159	147.0338
	DBO	5.467078	0.723774	4.935106	8.073513
	SABO	7.414	0.442234	6.832093	8.704741
	SCSO	6.973817	0.616379	6.154244	8.072619
	SO	2.1036632	0.4884498	1.2576818	3.1305641
	DTHSO	5.002573	0.336522	4.322848	6.107432
F6	PSO	6.35 × 10^−10^	1.34 × 10^−9^	9.24 × 10^−12^	6.14 × 10^−9^
	GWO	3.62 × 10^−6^	1.24 × 10^−6^	1.55 × 10^−6^	6.49 × 10^−6^
	WOA	0.002019	0.004102	0.000199	0.019776
	DBO	2.31 × 10^−23^	8.34 × 10^−23^	6.63 × 10^−30^	4.52 × 10^−22^
	SABO	0.044301	0.089992	0.000408	0.283947
	SCSO	0.050203	0.138549	2.38 × 10^−7^	0.505572
	SO	1.002 × 10^−14^	3.614 × 10^−14^	5.11 × 10^−22^	1.777 × 10^−13^
	DTHSO	1.4 × 10^−12^	2.22 × 10^−12^	7.53 × 10-16	1.02 × 10^−11^
F7	PSO	0.002595	0.001898	0.000397	0.008926
	GWO	0.000609	0.000427	9.96 × 10-5	0.001877
	WOA	0.002982	0.003414	0.000123	0.015604
	DBO	0.000979	0.000631	7.1 × 10^−5^	0.002622
	SABO	0.000177	0.000154	1.19 × 10^−5^	0.000602
	SCSO	0.000137	0.000285	1.23 × 10^−6^	0.001529
	SO	0.0002989	0.0002446	3.4 × 10^−5^	0.0009868
	DTHSO	0.000143	9.87 × 10-05	5.39 × 10^−6^	0.000431
F8	PSO	−3096.79	288.6078	−3854.25	−2432.97
	GWO	−2743.88	281.8803	−3298.59	−2220.43
	WOA	−3399.65	564.8312	−4189.78	−2451.76
	DBO	−3483.12	461.9972	−4188.44	−2522.59
	SABO	−1752.48	169.5616	−2290.32	−1493.77
	SCSO	−2578.91	308.291	−3143.56	−2040.75
	SO	−3399.49	410.44483	−4189.829	−2161.412
	DTHSO	−3588.87	292.6576	−4071.39	−3003.93
F9	PSO	6.311851	2.836308	1.989918	13.92943
	GWO	0.716722	1.688813	0.00	6.30707
	WOA	2.190373	9.516246	0.00	50.33645
	DBO	1.094453	4.183475	0.00	17.90923
	SABO	0	0	0	0
	SCSO	0	0	0	0
	SO	0	0	0	0
	DTHSO	0	0	0	0
F10	PSO	8.19 × 10^−6^	6.06 × 10^−6^	1.2 × 10^−6^	2.26 × 10^−5^
	GWO	6.84 × 10^−15^	1.45 × 10^−15^	4 × 10^−15^	7.55 × 10^−15^
	WOA	3.76 × 10^−15^	2.79 × 10^−15^	4.44 × 10^−16^	7.55 × 10^−15^
	DBO	4.44 × 10^−16^	0	4.44 × 10^−16^	4.44 × 10^−16^
	SABO	4 × 10^−15^	0	4 × 10^−15^	4 × 10^−15^
	SCSO	4.44 × 10^−16^	0	4.44 × 10^−16^	4.44 × 10^−16^
	SO	4.441 × 10^−16^	0	4.441 × 10^−16^	4.441 × 10^−16^
	DTHSO	4.44 × 10^−16^	0	4.44 × 10^−16^	4.44 × 10^−16^
F11	PSO	0.09904	0.04232	0.019701	0.211569
	GWO	0.018567	0.01878	0	0.057892
	WOA	0.056709	0.119564	0	0.436088
	DBO	0.014353	0.033802	0	0.137842
	SABO	0.005436	0.029776	0	0.163089
	SCSO	0	0	0	0
	SO	0	0	0	0
	DTHSO	0	0	0	0
F12	PSO	1.53 × 10^−10^	7.41 × 10^−10^	3.1 × 10^−13^	4.07 × 10^−9^
	GWO	0.008348	0.012343	2.31 × 10^−7^	0.041028
	WOA	0.008671	0.010178	0.000297	0.031783
	DBO	1.17 × 10^−24^	4.3 × 10^−24^	2.59 × 10^−30^	2.11 × 10^−23^
	SABO	0.043332	0.035159	0.001848	0.137659
	SCSO	0.026321	0.031426	1.84 × 10^−7^	0.160187
	SO	1.236 × 10^−15^	6.554 × 10^−15^	2.017 × 10^−20^	3.593 × 10^−14^
	DTHSO	8.28 × 10^−13^	1.34 × 10^−12^	8.69 × 10^−15^	6.75 × 10^−12^
F13	PSO	2.55 × 10^−9^	1.32 × 10^−8^	7.47 × 10^−13^	7.26 × 10^−8^
	GWO	0.013255	0.034356	1.41 × 10^−6^	0.100487
	WOA	0.055794	0.080734	0.000845	0.3936
	DBO	0.025435	0.048919	1.25 × 10^−28^	0.196254
	SABO	0.169119	0.118543	0.002289	0.426229
	SCSO	0.144537	0.139871	1.11 × 10^−6^	0.431344
	SO	0.0116692	0.026104	2.409 × 10^−20^	0.0988826
	DTHSO	0.023507	0.09711	8.78 × 10^−12^	0.496478
F14	PSO	1.130146	0.565887	0.998004	3.96825
	GWO	4.591674	4.023538	0.998004	12.67051
	WOA	4.295642	4.112566	0.998004	10.76318
	DBO	2.276595	2.557553	0.998004	10.76318
	SABO	3.896304	3.329865	1.031531	12.67081
	SCSO	3.871835	3.954056	0.998004	12.67051
	SO	2.5357039	3.0117596	0.9980038	10.763181
	DTHSO	1.262551	0.685995	0.998004	2.982105
F15	PSO	0.003209	0.006853	0.000307	0.020363
	GWO	0.008244	0.013001	0.000309	0.05662
	WOA	0.000553	0.000255	0.000308	0.001377
	DBO	0.000771	0.000346	0.000307	0.001333
	SABO	0.000905	0.001796	0.000316	0.010149
	SCSO	0.000547	0.000399	0.000308	0.001595
	SO	0.0053913	0.0087844	0.0003075	0.0225533
	DTHSO	0.000311	3.94 × 10^−6^	0.000307	0.000323
F16	PSO	−1.03163	6.05 × 10^−16^	−1.03163	−1.03163
	GWO	−1.03163	1.76 × 10^−8^	−1.03163	−1.03163
	WOA	−1.03163	1.17 × 10^−9^	−1.03163	−1.03163
	DBO	−1.03163	6.32 × 10^−16^	−1.03163	−1.03163
	SABO	−1.02525	0.012055	−1.03163	−0.98597
	SCSO	−1.03163	7.84 × 10^−10^	−1.03163	−1.03163
	SO	−1.031628	5.904 × 10^−16^	−1.031628	−1.031628
	DTHSO	−1.03163	5.3 × 10^−16^	−1.03163	−1.03163
F17	PSO	0.397887	0	0.397887	0.397887
	GWO	0.397895	2.7 × 10^−5^	0.397887	0.398037
	WOA	0.397894	1.54 × 10^−5^	0.397887	0.397961
	DBO	0.397887	0	0.397887	0.397887
	SABO	0.429105	0.053091	0.397924	0.588487
	SCSO	0.397887	4.69E-08	0.397887	0.397888
	SO	0.3978874	0	0.3978874	0.3978874
	DTHSO	0.397887	0	0.397887	0.397887
F18	PSO	3	1.68 × 10^−15^	3	3
	GWO	3.000034	5.53 × 10^−5^	3	3.000225
	WOA	3.000045	7.69 × 10^−5^	3	3.000341
	DBO	3	2.86 × 10^−15^	3	3
	SABO	4.58353	4.544002	3.000243	26.43964
	SCSO	3.000009	1.08 × 10^−5^	3	3.000038
	SO	11.1	24.715415	3	84
	DTHSO	3	9.55 × 10^−16^	3	3
F19	PSO	−3.83701	0.141133	−3.86278	−3.08976
	GWO	−3.86184	0.002065	−3.86278	−3.8549
	WOA	−3.85786	0.007432	−3.8627	−3.8231
	DBO	−3.86199	0.002405	−3.86278	−3.8549
	SABO	−3.63445	0.21976	−3.85595	−2.97954
	SCSO	−3.86065	0.003528	−3.86278	−3.8549
	SO	−3.862257	0.0019996	−3.862782	−3.854901
	DTHSO	−3.86278	2.56 × 10^−15^	−3.86278	−3.86278
F20	PSO	−3.29029	0.053475	−3.322	−3.2031
	GWO	−3.26275	0.071901	−3.32199	−3.11526
	WOA	−3.1885	0.141879	−3.32168	−2.63803
	DBO	−3.23231	0.110709	−3.322	−2.91606
	SABO	−3.24041	0.124298	−3.32092	−2.91461
	SCSO	−3.17712	0.220596	−3.32199	−2.26724
	SO	−3.253135	0.0733724	−3.321995	−3.132697
	DTHSO	−3.24273	0.057005	−3.322	−3.2031
F21	PSO	−5.52228	3.451826	−10.1532	−2.63047
	GWO	−8.97841	2.44141	−10.1528	−2.68255
	WOA	−7.52317	2.926188	−10.1525	−2.62409
	DBO	−7.3624	2.689533	−10.1532	−2.63047
	SABO	−4.88783	0.574508	−6.62134	−3.409
	SCSO	−5.68644	2.207265	−10.1532	−0.88199
	SO	−9.593368	2.142856	−10.1532	−0.880982
	DTHSO	−5.31576	1.387429	−10.1532	−2.63047
F22	PSO	−6.17398	3.390893	−10.4029	−2.75193
	GWO	−10.4012	0.000611	−10.4025	−10.4
	WOA	−7.49996	3.452079	−10.402	−1.8352
	DBO	−8.02273	3.057761	−10.4029	−1.83759
	SABO	−4.83898	0.550266	−5.08625	−2.66295
	SCSO	−6.78202	2.637799	−10.4029	−2.7659
	SO	−8.748751	3.0586222	−10.40294	−2.765897
	DTHSO	−5.98244	2.30892	−10.4029	−2.7659
F23	PSO	−6.22872	3.903871	−10.5364	−1.67655
	GWO	−10.5344	0.001083	−10.5363	−10.5314
	WOA	−6.8525	3.314703	−10.5348	−1.85892
	DBO	−8.8277	2.69506	−10.5364	−2.42173
	SABO	−4.85353	1.101254	−9.5443	−2.1511
	SCSO	−6.29194	2.805714	−10.5364	−0.94888
	SO	−8.697027	3.4025103	−10.53641	−1.85948
	DTHSO	−7.46185	3.206559	−10.5364	−2.42173

## Data Availability

The data that support the findings of this study are available from the corresponding author upon request. There are no restrictions on data availability.
